# Abundance of Maternal Mitochondrial Genome Is Dispensable up to the Mitochondrial Genome Activation in Post‐Implantation Embryos

**DOI:** 10.1096/fj.202501179R

**Published:** 2025-09-09

**Authors:** Miki Shavit, Marie Vancová, Jan Jedlicka, Tomáš Bílý, Mushrek Mahrouk, Jan Cendelin, Kateřina Grygarova, Kristýna Popelková, Zdeněk Tůma, Gabriela Pribanova, Jitka Kuncová, Jan Nevoral

**Affiliations:** ^1^ Faculty of Medicine in Pilsen, Biomedical Center Charles University Prague Czech Republic; ^2^ Second Faculty of Medicine, Department of Histology and Embryology Charles University Prague Czech Republic; ^3^ Institute of Parasitology, Biology Centre Czech Academy of Sciences České Budějovice Czech Republic; ^4^ Faculty of Science University of South Bohemia České Budějovice Czech Republic; ^5^ Faculty of Medicine in Pilsen, Department of Physiology Charles University Pilsen Czech Republic; ^6^ Faculty of Medicine in Pilsen, Department of Pathological Physiology Charles University Pilsen Czech Republic; ^7^ Third Faculty of Medicine, Department of Histology and Embryology Charles University Prague Czech Republic; ^8^ Faculty of Medicine in Pilsen, Department of Histology and Embryology Charles University Pilsen Czech Republic

**Keywords:** embryo, fertilization, mitochondrial, mitochondrion, oocyte, transcription factor A

## Abstract

Mitochondria in the egg are suggested to be crucial for the onset of new life. However, there is ambiguous knowledge about the necessity for fertilization and early embryonic development. Therefore, we created a conditional *Tfam* knockout (*Tfam* ^loxP/loxP^; Zp3‐Cre) to produce *Tfam* ^null^ oocytes for investigation of the mitochondrial abundance in oocytes and early embryos. This created mtDNA‐depleted eggs, although the abundance of mitochondria did not change. Despite decreased mitochondrial membrane potential, *Tfam* ^null^ oocytes matured and were fertilized, which led to embryo formation. These *Tfam* ^null^ eggs were developed into mtDNA‐deficient blastocysts. Both TFAM and mtDNA appear to be dispensable for the success of embryo implantation. *Tfam* expression and mtDNA replication rescue the mtDNA‐deficient embryo after implantation, enabling passage through a post‐implantation bottleneck, and allowing survivor embryos to develop into healthy individuals. Our findings highlight the uncoupled relationship between mtDNA replication and mitochondrial abundance in the growing oocyte and show the importance of the oocyte bulk mtDNA for successful mitochondrial activation in post‐implantation embryos.

## Graphical abstract

A mouse model of *Tfam* conditional knock‐out (*Tfam* ^loxP/loxP^; Zp3‐Cre) produces mtDNA‐depleted eggs. An abundance of mitochondrial mass is maintained through fission during oocyte growth and enables egg fertilization and blastocyst formation. Although embryonic genome activation may compensate for the maternal knock‐out in the two‐cell stage, the paternal *Tfam* allele is expressed only in the post‐implantation embryo. Expression of *Tfam* leads to the activation of the mitochondrial genome in the post‐implantation embryo, and mitochondrial abundance is restored in the somatic tissues of F1 animals. The ability of mitochondrial biogenesis re‐initiation is limiting for the surveillance of post‐implantation embryos.

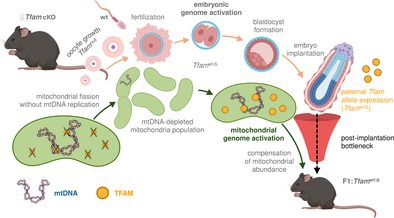

## 1 Introduction

The abundance and quality of the mitochondrial pool in the egg are considered crucial markers of developmental competence and successful conception [1]. This assumption has been prevalent for decades, and the consecutively accumulated knowledge of mitochondrial dynamics has further supported the dogma of mitochondrial necessity for the oocyte [2, 3]. Accordingly, together with the massive expansion in volume, the growing oocyte enlarges the mitochondrial population, including replication of mitochondrial DNA (mtDNA). Thus, a fully grown mature oocyte, an egg, contains 37 crucial genes for oxidative phosphorylation housed in more than 100 000 copies of mtDNA [4, 5]. It is believed that semi‐autonomous machinery covers the energy demand for chromatin remodeling of the germinal vesicle and meiotic maturation, leading to the formation of a fertile oocyte arrested at metaphase II [6, 7, 8].

In contrast to the traditional dogma of mitochondrial powerhouse, we underline the heritage of the mitochondrial genome as playing a major role in the egg; this assumption is based on the fact of inheritance down the maternal lineage [9] due to sophisticated autophagy of sperm mitochondria in the zygote [10]. Indeed, growing evidence indicates that oxidative phosphorylation is not critical for oocyte growth or early embryos [11, 12]. Oxidative phosphorylation is severely reduced during early oogenesis, and the mitochondrial electron transport chain is remodeled to eliminate reactive oxygen species (ROS) production [11]. On the other hand, replication of mtDNA seems to be essential in growing oocytes, achieving the minimum of mtDNA copy number required for successful embryonic development [4]. The copy number seemingly loses necessity once the threshold is exceeded; accordingly, there is no association between mtDNA copy number and the outcome of embryo transfer [13], nor a correlation with embryo aneuploidy [14].

Several ambiguous facts led us to design a model of mitochondria‐depleted oocyte and to study mitochondrial contribution across milestones of embryonic development. For this purpose, we have chosen the engineering of gene coding transcription factor A, mitochondrial (TFAM), due to its necessity for both mtDNA replication and transcription [15]. Accordingly, we assume an essential role of TFAM in growing oocytes for the establishment of the mitochondrial genome via progressive replication of mtDNA. For this reason, we designed a conditional knockout (cKO) of *Tfam* in growing oocytes and hypothesized mitochondrial‐depleted eggs. After fertilization with wild‐type (wt) sperm, we suggested a compensation of oocyte TFAM deficiency through the paternal *Tfam* allele expression, once the embryonic genome activation (EGA) occurs at the two‐cell stage [16]. Our study reveals the independence of mitochondrial fission and mtDNA replication in the growing oocyte and describes a noteworthy model of a mtDNA‐depleted egg. Our knowledge renders new insight into reproductive medicine and assisted reproductive technologies, and sheds further light on the often ambiguous findings regarding the importance of oocyte mitochondria for reproductive events.

## 2 Methods

### 2.1 Mice

All animal procedures were conducted in accordance with Act No. 246/1992 Coll., on the Protection of Animals against Cruelty, under the supervision of the Animal Welfare Advisory Committee at Charles University, Faculty of Medicine in Pilsen, and approved by the Animal Welfare Advisory Committee at the Ministry of Education, Youth and Sports of the Czech Republic (MSMT‐33798/2021‐4). Mouse strain possessing loxP sites flanking exon 6–7 of the *Tfam* gene was purchased from The Jackson Laboratory (B6.Cg‐*Tfam* ^tm1.1Ncdl^/J, #026123). To generate cKO *Tfam* ^loxP/loxP^; *Zp3*‐Cre mice, female mice carrying the *Tfam* floxed alleles were crossed with *Zp3*‐Cre females (Jackson Laboratories; C57BL/6‐Tg(Zp3‐cre)93Knw/J, #003651). Cre‐positive females were used as donors of *Tfam*‐deficient oocytes, along with oocytes provided by Cre‐negative females as wt control. Mice were housed in a 12–12 h light–dark cycle, with constant temperature and with a commercial pellet diet and water provided *ad libitum*. Genotyping for LoxP and Cre was carried out, using PCR amplification of tail snip genomic DNA. Primers for *Tfam* ^*LoxP*^ (Forward: 5′‐ATCATTGGAGGTTATAACATGAATTG‐3′, Reverse: 5′‐GTTACCTATTGAACGCCCTACGAGAG‐3′), and *Zp3*‐Cre (Forward: 5′‐GCGGTCTGGCAGTAAAAACTATC‐3′, Reverse: 5′‐GTGAAACAGCATTGCTGTCACTT‐3′, Internal control Forward: 5′‐CTAGGCCACAGAATTGAAAGATCT‐3′, Internal control Reverse: 5′‐GTAGGTGGA AATTCTAGCATCATC C‐3′) were used at 20 pMol, using FastMix French PCR beads (Bulldog Bio, #25401), following the manufacturer's protocol. DNA was stained, using SYBR Safe DNA gel stain (S33102; ThermoFisher Scientific, Waltham, MA, USA) and scanned on a gel station (Universal Hood II; Bio‐Rad, France).

### 2.2 Fertility Trials

Sexually mature wt and cKO 8‐ to 12‐week‐old female mice were mated overnight to C57Bl/6 (wt) males. The number of matings of hormonally stimulated females was recorded and shown as the conception rate. The number of pups of 1st, 2nd, and 3rd litters was recorded and presented as a litter size. Moreover, female fertility was assessed via hormonal responsiveness, and the number of ovulated oocytes was recorded.

### 2.3 Collection of Mouse Oocytes

8‐ to 12‐week‐old female mice were used as oocyte donors. Germinal vesicle (GV) oocytes were isolated from the ovaries of donors after 44–48 h of PMSG (pregnant mare serum gonadotropin) administration. For the collection of in vivo‐ovulated (IVO) matured oocytes (i.e., eggs), PMSG administration was followed by hCG (human chorionic gonadotropin) 48 h later; 15–16 h post‐hCG injection, ovulated cumulus‐oocyte complexes (COCs) were flushed from the fallopian tube. To remove cumulus cells, COCs were treated with 0.1% hyaluronidase (Sigma‐Aldrich, #H3506) in hepes‐buffered human tubal fluid (HTF) medium, modified with 0.1% bovine serum albumin (BSA; mHTF‐hepes), for 5 min at 37°C. These oocytes and cumulus cells were used henceforward.

### 2.4 In Vitro Fertilization Assay (IVF)

14‐week‐old male mice were euthanized by cervical dislocation, and epididymal cauda were dissected. Spermatozoa were then isolated in mHTF, supplemented with 0.4% BSA (mHTF). Next, in vitro capacitation was allowed for 30 min under 5% CO_2_ and 37°C under oil. Spermatozoa were then stained with MitoTracker Deep Red FM (100 nM; M22426, Invitrogen) for the next 30 min under the same conditions. Meanwhile, cumulus‐oocyte complexes were isolated from the ampulla of PMSG‐hCG‐stimulated females. Complexes were then co‐incubated with the capacitated sperm for 5.5 h in mHTF medium, under the same conditions applied for capacitation. Thereafter, zygotes were denuded from the rest of the cumulus cells and cultured in potassium simplex optimization medium (KSOM), modified with 0.1% BSA (mKSOM), for 4 days under the conditions described above. Cleavage and blastocyst rates were recorded 24 h and 96 h later, respectively.

### 2.5 Parthenogenetic Activation of Mouse Oocytes

Matured IVO oocytes were incubated in mKSOM as described above. For the preparation of activating medium, the mKSOM was supplemented with 2 mM EGTA, 10 mM SrCl_2_, and 5 μg/mL cytochalasin B, for 5.5 h at 37°C and 5% CO_2_. Cycloheximide (CHX; 15 μg/mL) was used for the inhibition of proteosynthesis during oocyte activation. Afterwards, embryos were moved into pure mKSOM and cultured for 24 h and 96 h to cleaved embryos and blastocyst stages, respectively. Transcription in pre‐EGA parthenotes was inhibited using α‐amanitin (11 μg/mL), an RNA polymerase II and III inhibitor, for 26 h following the activation.

### 2.6 Post‐Implantation Embryo Collection

Wt and *Tfam* cKO females were naturally mated with *Tfam* ^+/−^ males. Vaginal plug appearance was recorded every morning. 12 to 15 days later, fetuses were isolated *post mortem*, and the ratio of abortus was recorded. Samples were collected, and genotyping was performed as described above.

### 2.7 The Assessment of mtDNA Copy Number

Matured IVO oocytes were pooled from two female donors and placed in oocyte lysis buffer, consisting of 50 mM Tris–HCl, pH 8.5, with 0.5% Tween 20 and 200 ng mL^−1^ proteinase K. Samples were incubated at 55°C for 30 min, followed by incubation at 95°C for 15 min, and stored at −20°C until use. Muscle tissues were placed in ATL buffer from Qiagen, homogenized via bead beating with glass beads, and total genomic and mitochondrial DNA was extracted using a DNeasy Blood & Tissue Kit (Qiagen, #69504) as per the manufacturer's instructions. Oocyte mtDNA copy number was calculated by absolute quantification, using primers specific for mouse mtDNA [17] and a standard curve using a PCR‐generated template. All samples and standards were measured in technical triplicates. Quantitative real‐time PCR (qPCR) was performed on a CFX96 Touch Real‐Time PCR Detection System (Bio‐Rad), using the following protocol: pre‐incubation at 95°C for 5 min (1 cycle); denaturation at 95°C for 10 s, annealing and extension at 60°C for 30 s, repeated for 40 cycles, followed by melt curve analysis. No PCR product was observed in the no‐template control after 40 PCR cycles, and the specificity of the primers was confirmed as a single melt peak and single band when electrophoresed on a 3% agarose gel (not shown). qPCR efficiency calculated from the slope was between 95% and 105% with co‐efficiency of reaction *R* ^2^ = 0.98–0.999. For muscle tissue, the relative fold of mtDNA to genomic DNA was established via qPCR, using primers specific for mouse mtDNA and genomic DNA [17] using DBdirect PCR SYBR Mix (DIANA Biotechnologies; DB‐1271) and the same settings for the run as described above.

### 2.8 Isolation and qRT‐PCR of mtRNA Transcripts

Matured IVO oocytes and cumulus cells were pooled from two female donors and placed in 20 μL TRIzol Reagent (Invitrogen) and total RNA was extracted as per the manufacturer's instructions. Single‐stranded cDNA was generated from purified total RNA using a High‐Capacity cDNA Reverse Transcription Kit (Applied Biosystems; #4368814), and the relative fold expression of mtRNA transcripts normalized to beta actin mRNA was established via qPCR, using PerfeCTa SYBR Green FastMix (Quantbio) on a CFX96 Touch Real‐Time PCR Detection System (Bio‐Rad), using the same protocol as described above. Muscle tissues (collected as described below) were placed in TRIzol Reagent, homogenized via bead beating with glass beads, and total RNA was extracted as per the manufacturer's instructions. The relative fold expression of mtRNA (mt‐Atp6, mt‐Nd1, mtNd4l) and nuclear transcripts (Atp5g3, Ndufv1) was normalized to β‐actin mRNA and quantified via “one‐step” RT‐qPCR using DBdirect RT‐PCR SYBR Mix (DIANA Biotechnologies; DB‐1265) on a CFX96 Touch Real‐Time PCR Detection System (Bio‐Rad), as described above. Primers for β‐actin [18] and mitochondrial genes [19] were described previously.

### 2.9 Transmission Electron Microscopy

Oocytes were fixed in 2.5% glutaraldehyde in 0.1 M phosphate buffer (PB) for 1 h at room temperature, followed by storage at 4°C. Subsequently, the samples were washed three times in PB with 4% glucose, 15 min each. Samples were then incubated in 1% OsO_4_ for 1 h at 4°C, then in 1.5% K_4_Fe(CN)_6_ (31 254, Sigma‐Aldrich) for 1 h at 4°C, washed three times, and dehydrated through a graded acetone series (30%, 50%, 75%, 80%, 90%, and 95%; 15 min each step) at RT. Samples were infiltrated in 25%, 50%, 75% Spi‐Pon 812 kit (SPI) followed by overnight incubation in pure resin. Samples were polymerized at 60°C for 48 h. Ultrathin sections were stained with saturated ethanolic uranyl acetate for 30 min and lead citrate for 20 min, and carbon coated. Oocyte mitochondria were imaged using a JEOL 1400 Flash transmission electron microscope equipped with an EMSIS Xarosa CMOS camera. Images with a resolution of 5120 × 3840 pixels and a pixel size of 2 nm were analyzed in Microscopy Image Browser [20]. Mitochondria were manually selected, and segmentation was subsequently performed using the Segment Anything 2 model developed by Meta AI [21], which enables class‐agnostic segmentation through promptable vision foundation models. Images for quantitative analysis were acquired randomly from 10 to 13 areas per oocyte, with each area measuring 78.64 μm^2^. Three independent biological replicates (i.e., three female donors) were analyzed, each comprising at least 3 oocytes per female. Mitochondrial area and number of mitochondria per μm^2^ were analyzed.

### 2.10 Immunocytochemistry

Oocytes and embryos were fixed in 4% paraformaldehyde in PBS with 0.1% polyvinyl‐alcohol (PVA), 30 min at room temperature. Oocytes and embryos were permeabilized in PBS containing 0.1% Triton X‐100 for 15 min, and blocked in 1% BSA in PBS with 0.01% Tween 20 for 15 min afterwards. The oocytes were incubated with anti‐TFAM (Abcam, ab252432; 1:200) overnight at 4°C. Afterwards, washing and 1 h incubation with fluorescein‐conjugated secondary antibodies (1:200) were carried out. Along with subsequent washing, β‐actin was stained using Alexa 594‐conjugated Phalloidin (Thermo Fisher Scientific, USA; 1:200). Oocytes and embryos were mounted onto slides in a Vectashield medium with 4′6′‐diami‐dino‐2‐phenylindole (DAPI; Vector Laboratories Inc., Burlingame, CA, USA). Specimens were imaged using the Nikon AX R scanning confocal microscope, equipped with a high‐speed resonant scanner (Nikon, Japan) and NIS Elements Advanced Research software (Laboratory Imaging Inc., Czech Republic). Maximum intensity projections of Z‐stacks were subjected to measurement of “integrated density” (IntDen) of appropriate color channels using ImageJ software (NIH, Bethesda, CA, USA).

### 2.11 Measurement of Mitochondrial Membrane Potential (ΔΨm)

Mitochondrial mass was stained in the IVO oocytes of wt and *Tfam* cKO females using various probes in accordance with different dye behavior summarized earlier [22]. Firstly, vital staining of mitochondria with 200 nM of MitoTracker Green, FM (M7514, Invitrogen) was used, combined with 20 nM of tetramethyl rhodamine methyl ester (TMRM; 20 036, Invitrogen) for tracking of mitochondrial membrane potential (ΔΨm) changes, as described previously [23]; carbonyl cyanide 3‐chlorophenylhydrazone was used as an uncoupler to depolarize the mitochondrial membrane in accordance with the manufacturer's instructions. Separately, 5 ng/mL of 5,5′,6,6′‐Tetrachloro‐1,1′,3,3′‐tetraethylbenzimidazolocarbocyanine iodide (JC‐1; T4069, Merck, USA) was applied for another assessment of ΔΨm. All probes were dissolved in mHTF‐hepes (1:1000) and oocytes were kept for 30 min at 37°C. Live oocytes were washed in mHTF‐hepes and imaged using an upright Nikon Ci microscope (Nikon, Japan) and device (Optika, Italy). The IntDen of MitoTracker and TMRM dyes was measured using the ImageJ software (NIH, USA) and expressed as a fold change related to the wt. Change of JC‐1 IntDen was assessed by the ratio of monomer (green) and aggregate (red).

### 2.12 Muscle Strength Measurement

Muscle strength was measured in mice aged 12–14 weeks using Grip Strength Test apparatus (Bioseb, USA). The mouse tail was held by the experimenter's hand, and the mouse was allowed to grasp a metal grid of the device with both its forepaws. Thereafter, the experimenter began to pull gently on the tail, slowly increasing the strength. The apparatus recorded the power being set to keep the maximum value on the display. The maximum value was registered after the mouse let go of the grid. The measurement was repeated to obtain 10 values, which were then averaged. Values from trials during which the mouse also grasped the grid with its hindpaws or teeth, or only with one of its forepaws, were not used.

### 2.13 High‐Resolution Respirometry

Following the muscle strength test, animals were euthanized and freshly extracted samples of quadriceps femoris muscle were weighed, homogenized using PBI‐Shredder (Oroboros, Austria) and placed into 4 pre‐calibrated oxygraphs (O2k, Oroboros, Austria), each containing 2 chambers with a volume of 2 mL in MiR05 medium (EGTA 0.5 mmol/L, MgCl_2_·6H2O 3 mmol/L, potassium lactobionate 60 mmol/L, taurine 20 mmol/L, KH_2_PO_4_ 10 mmol/L, HEPES 20 mmol/L, sucrose 110 mmol/L, fatty acid free bovine serum albumin 1 g/L, pH 7.0) at 37°C. After equilibration, measurements were initiated using a standardized SUIT (Substrate‐Uncoupler‐Inhibitor‐Titration) protocol, which begins with titration of malate (M; 0.1 mmol/L) and palmitoyl‐L‐carnitine (Pcar; 0.04 mmol/L) to evaluate the LEAK state—non‐phosphorylating respiration compensating for proton leak across the inner mitochondrial membrane; adenosine diphosphate (ADP; 5 mmol/L) – phosphorylating state with activated metabolism of fatty acids; cytochrome c (c; 10 μmol/L) – to evaluate the integrity of the outer mitochondrial membrane; glutamate (G; 10 mmol/L) and pyruvate (P; 5 mmol/L), both providing electrons to Complex I; succinate (S; 50 mmol/L) – a Complex II substrate; FCCP (Carbonyl cyanide p‐trifluoro‐methoxyphenyl hydrazone; 0.05 μmol/L, gradual titration) to evaluate the Electron Transport System Capacity (ETSC) – maximal respiration after oxidation and phosphorylation uncoupling; rotenone (ROT; 0.5 μmol/L) inhibitor of Complex I, and antimycin A (AMA; 2.5 μmol/L) inhibitor of Complex III, to measure residual oxygen consumption. The activity of Complex IV was determined by titration of TMPD (N,N,N′,N′‐Tetramethyl‐p‐phenylenediamine dihydrochloride; artificial substrate of complex IV; 0.5 mmol/L), ascorbate (reducing agent for high auto‐oxidation of TMPD; 2 mmol/L), and azide (AZD; Complex IV inhibitor; 100 mmol/L). All samples were kept on ice in BIOPS preservation solution (CaK_2_EGTA 2.77 mol/L, K_2_EGTA 7.23 mmol/L, imidazole 20 mmol/L, taurine 20 mmol/L, 50 mmol/L, MES hydrate, 50 mmol/L dithiothreitol, MgCl_2_·6H_2_O 6.56 mmol/L, Na_2_ATP 5.77 mmol/L, Na_2_phosphocreatine 15 mmol/L, pH 7.1) at all times. The data were evaluated using DatLab software, version 7.4, normalized to wet tissue mass or citrate synthase activity.

### 2.14 Citrate Synthase Activity

The medium for determining citrate synthase activity consists of mixing 0.1 mmol/L 5,5‐dithio‐bis‐(2‐nitrobenzoic) acid, 0.25% Triton‐X, 0.5 mmol/L oxaloacetate, 0.31 mmol/L acetyl coenzyme A, 5 μmol/L EDTA, 5 mmol/L triethanolamine hydrochloride, and 0.1 mol/L Tris–HCl, pH 8.1; 20 μL of the mixed and homogenized content of the oxygraph cell was added to 180 μL of the medium in a 96‐well plate. The rate of absorbance change (RAC) was measured spectrophotometrically at 412 nm and 30°C after 200 s.

### 2.15 Statistics

The data were analyzed using GraphPad Prism 8.1.1 (GraphPad Software Inc., San Diego, CA, USA). Based on Shapiro–Wilk's normality distribution tests, differences in quantitative variables were tested using either the *t*‐test or Mann–Whitney *U* test. Categorical variables were tested using the Chi‐squared test. *p*‐values < 0.05 were considered statistically significant and indicated with asterisks in graphs. Normally and non‐normally distributed data are expressed as means and medians, respectively.

## 3 Results

### 3.1 Conditional *Tfam* Knockout Females Provide a Model of mtDNA‐Depleted Eggs

**FIGURE 1:**
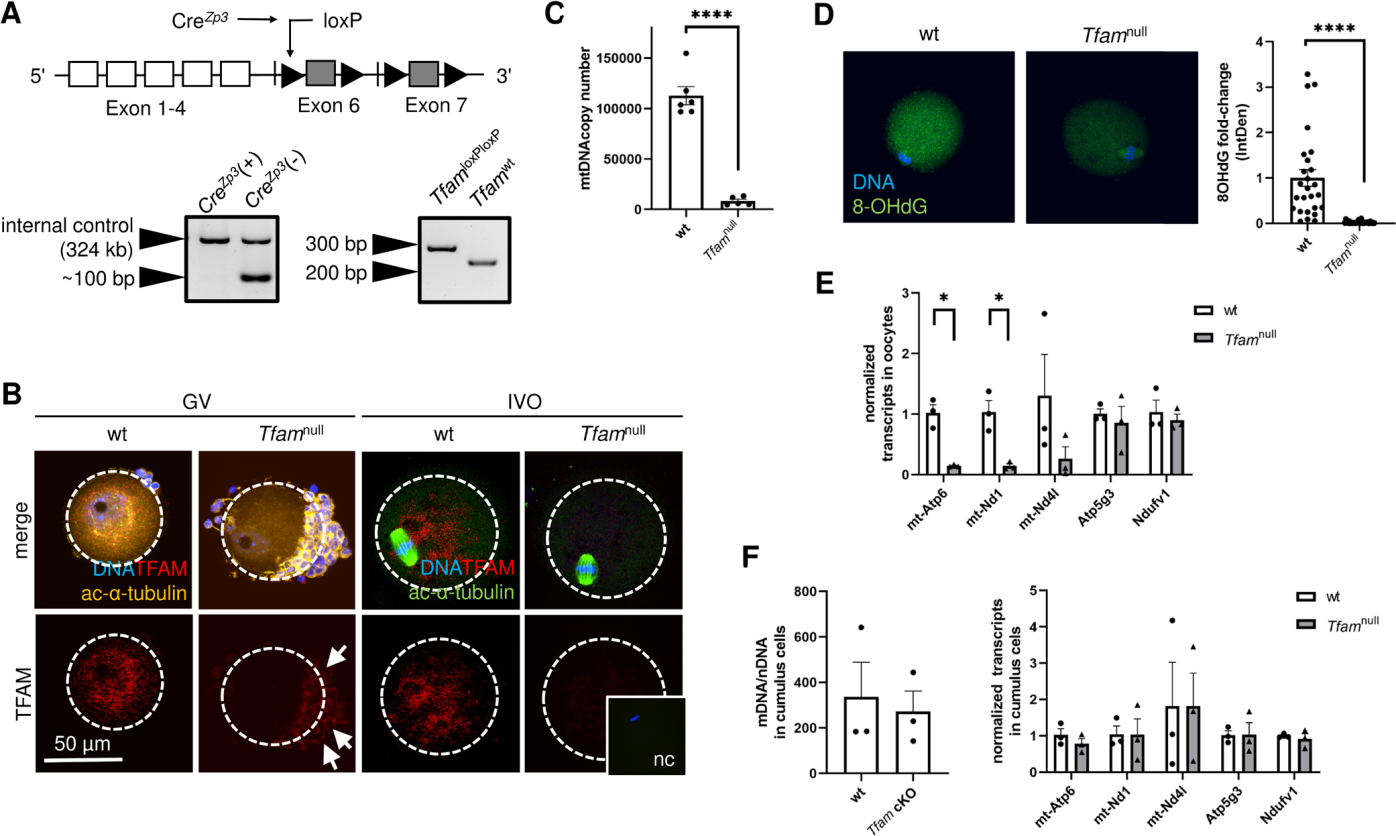
Genesis and depiction of *Tfam* ^null^ oocytes. (A) Generation of mouse *Tfam* ^loxP/loxP^; *Zp3*‐Cre (cKO) females as donors of *Tfam* ^null^ oocytes. *Tfam* allele contains loxP sites (black triangle), flanking exon 6–7, and results in *Tfam* ^loxPloxP^ allele deletion by Cre recombinase in *Cre* ^*Zp3*^(+) females. *Cre* ^*Zp3*^(−); *Tfam* ^loxP/loxP^ females were used as wild‐type (wt) control. (B) Distribution of TFAM in immature (GV: germinal vesicle stage) and matured (IVO: in vivo‐ovulated) oocytes. At least 10 oocytes per genotype and stage were stained. Arrows point to the TFAM signal in granulosa cells accompanying *Tfam* ^null^ oocytes. The negative control (nc) is included when the primary antibody was omitted. (C) Mitochondrial (mt) DNA copy number in wt and *Tfam* ^null^ IVO oocytes. Columns represent mean ± S.E.M., and dots show pooled samples (two female donors each). Differences were tested using a paired *t*‐test (****p < 0.0001). (D) Quantification of mtDNA through immunostaining of 8‐hydroxy‐2′‐deoxyguanosine (8OHdG) in wt and *Tfam* ^null^ IVO oocytes. Columns represent mean ± S.E.M., and dots show individual oocytes. Differences were tested using an unpaired *t*‐test (****p < 0.0001). (E) Expression of mitochondrial (mt‐Atp6, mt‐Nd1, mtNd4l) and nuclear transcripts (Atp5g3, Ndufv1) in wt and *Tfam* ^null^ matured oocytes. (F) The mtDNA copy number assessment and gene expression in cumulus cells of wt and *Tfam* cKO females, surrounding wt and *Tfam* ^null^ oocytes, respectively. Gene expression levels were normalized to β‐actin expression; columns show mean ± S.E.M. of normalized fold expression values, and dots show pooled samples. Differences between mean dCt values of wt and *Tfam* ^null^ animals were tested, using a paired *t*‐test (*p < 0.05).

For the study of mitochondria in the oocyte, we created a conditional knockout of *Tfam* using Cre recombinase‐driven excision of the *Tfam* ^lox/lox^ gene, specifically in growing oocytes (Figure 1A). Indeed, *Tfam*‐deficient (*Tfam* ^null^) oocytes have no signal for TFAM protein in either stage: germinal vesicle (GV) and in vivo ovulated, i.e., immature oocytes in prophase I and matured metaphase II eggs, respectively (Figure 1B). According to our assumption, *Tfam* ^null^ eggs, destined for fertilization, showed a significant decline in mtDNA copies (Figure 1C). This observation was supported by the staining of 8‐hydroxy‐2′‐deoxyguanosine (8OHdG), an oxidized form of guanosine abundant in mtDNA (Figure 1D). In addition to mtDNA replication, we tested transcription of the mitochondrial genome and observed a significant decrease in key mitochondrial transcripts in *Tfam* ^null^ eggs (Figure 1E). Accordingly, we did not find changes in mtDNA copy number and nuclear transcripts for wt cumulus cells surrounding *Tfam* ^null^ eggs (Figure 1F). Based on the oocyte‐specific excision of *Tfam*, we achieved a model of mtDNA‐depleted eggs with unknown mitochondrial quality entering the reproductive process.

### 3.2 Uncoupled Mitochondrial Fission and mtDNA Replication in Growing Oocytes

**FIGURE 2:**
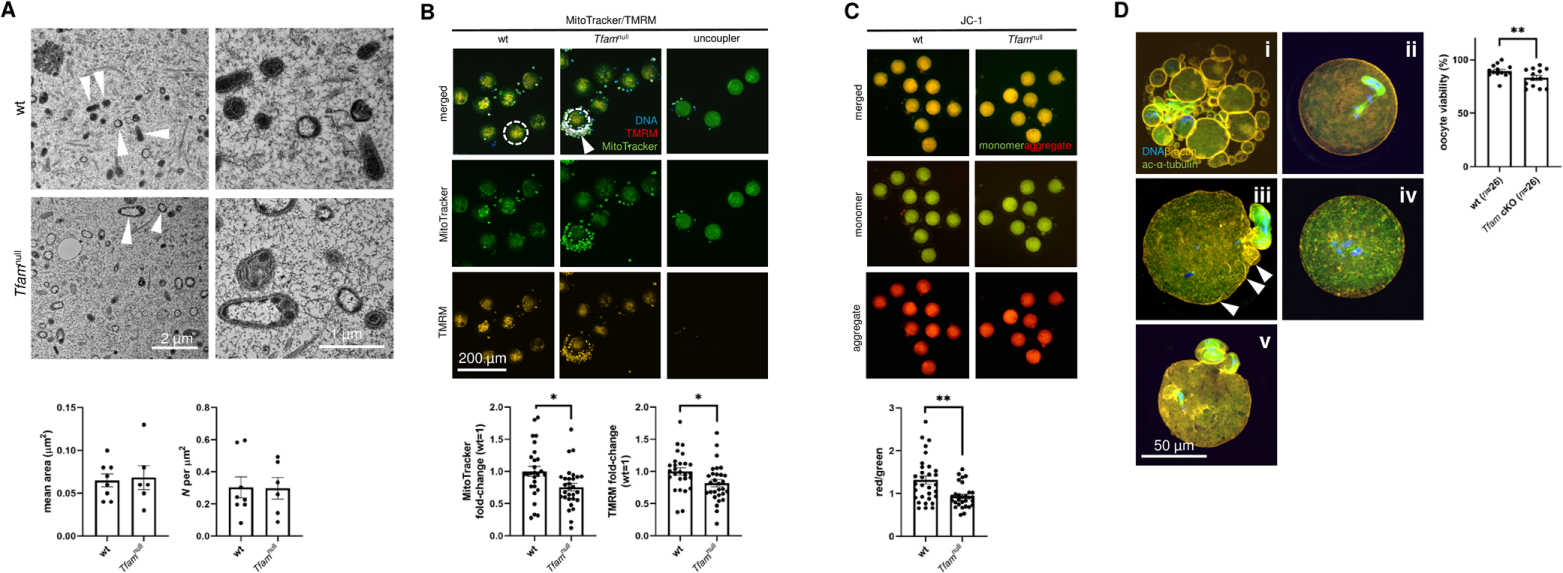
Analysis of mitochondrial mass in mtDNA‐depleted eggs. (A) Visualization and quantification of mitochondria in wt and *Tfam* ^null^ oocytes using transmission electron microscopy. Arrowheads point to mitochondria. Columns represent mean ± S.E.M., and dots show individual oocytes of three wt and *Tfam* cKO female donors. Differences were tested using an unpaired *t*‐test. (B) Mitochondrial staining and measurement of changed membrane potential of mitochondria (ΔΨm) in IVO oocytes of wt and *Tfam* ^null^, using MitoTracker Green and tetramethyl rhodamine methyl ester (TMRM), respectively. Circle shows the region of interest (ROI) to integrated density (IntDen) measurement; arrowhead points surrounding cumulus cells (out of the ROI). The IntDen is expressed as a fold change of MitoTracker Green and TMRM related to wt. Columns represent mean ± S.E.M., and dots show individual oocytes through three independent experiments. Differences were tested using the unpaired *t*‐test (*p < 0.05). Carbonyl cyanide 3‐chlorophenylhydrazone was used as an uncoupler to induce mitochondrial membrane depolarization. (C) The Δ4Ψm assessment using 5,5′,6,6′‐Tetrachloro‐1,1′,3,3′‐tetraethylbenzimidazolocarbocyanine iodide (JC‐1). Change is expressed as a ratio of monomer (green) and aggregate (red) of JC‐1 dye. Columns represent mean ± S.E.M., and dots show individual oocytes through three independent experiments. Differences were tested using the unpaired *t*‐test (**p < 0.01). (D) Assessment of oocyte viability as a ratio of viable and total flushed IVO oocytes. Different types of oocyte degeneration are demonstrated as follows: Fragmentation (i.e., apoptosis; i), inability to extrude the 1st polar body (ii), membrane blebbing (arrowheads) and aneuploidy (all chromosomes extruded to the polar body; iii), meiotic arrest (iv), and complete chromosomal misalignment (v). Columns represent mean ± S.E.M., and dots show individual trials. Numbers of female donors are noted in brackets. Differences were tested using a paired *t*‐test (**p < 0.01).

The dramatic decline of mtDNA content raised concerns about the mitochondrial fitness of *Tfam* ^null^ eggs. For this reason, we subjected eggs to investigation by transmission electron microscopy. Surprisingly, mtDNA‐depleted eggs contain equal mitochondrial mass (Figure 2A). Therefore, we assume most of the mitochondria do not bear mtDNA molecules. Nevertheless, mtDNA‐depleted mitochondria show impaired membrane potential (ΔΨm) assessed by vital staining with different fluorescent probes (Figure 2B,C). The uncoupler was used as a control of depolarized mitochondrial membranes, which avoids TMRM staining (Figure 2B). The decline in membrane potential likely occurred due to the insufficiency of mtRNA transcription (Figure 1E), limiting the generation of new electron transport chain complexes. The viability of ovulated eggs is also decreased, and we may assign different phenotypes (e.g., fragmentation, inability to extrude the 1st polar body, chromosome mis‐segregation; Figure 2D) to mitochondrial dysfunction. Taken together, we considered ambiguous mitochondrial status in *Tfam* ^null^ eggs and, ultimately, the fertility of carriers.

### 3.3 Mitochondria, Not Nucleus‐Encoded Burden, of *Tfam*‐Deficient Eggs Determines the Success of Pregnancy

**FIGURE 3:**
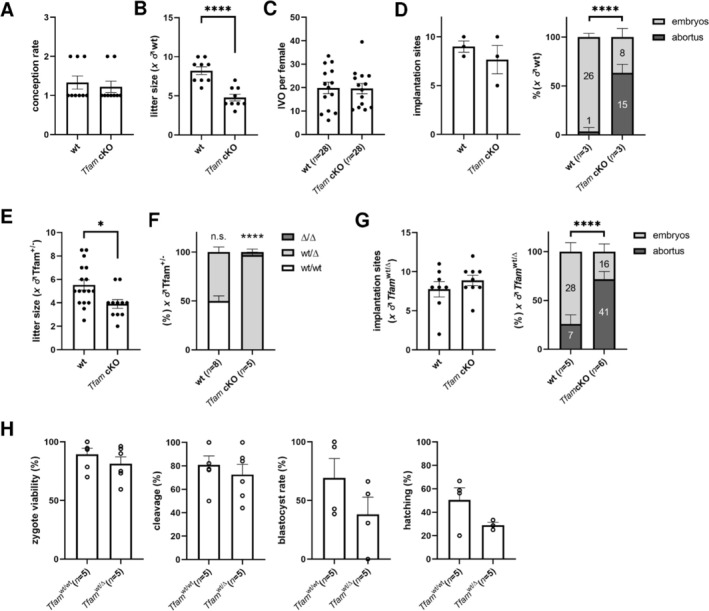
Reproductive fitness of carriers of mtDNA‐depleted eggs and success of *Tfam*‐insufficient embryos (A) Reproductive phenotype assessment via conception rate after natural mating with wild‐type (wt) males. Columns represent mean ± S.E.M., and dots show females. Differences were tested using a paired *t*‐test. (B) Record of the litter size after natural mating with wt males. Columns represent mean ± S.E.M., and dots show females. Differences were tested using a paired *t*‐test (****p ≤ 0.0001). (C) Hormonal responsiveness (i.e., IVO per female) of donors and oocyte viability. Columns represent mean ± S.E.M., and dots indicate independent trials. The numbers of animals are indicated in brackets. Differences were tested using a paired *t*‐test (**p < 0.01; ****p < 0.0001). (D) Implantation sites and rate of abortion after mating of wt and *Tfam* cKO females with wt males. Columns represent mean ± S.E.M., and dots indicate independent trials. Differences were tested using a paired *t*‐test. Stacked columns represent mean ± S.E.M. of embryos and abortus; number of pregnancies is indicated in brackets. Differences were tested using the Chi‐square test (****p < 0.0001). (E) Litter size of wt and *Tfam* cKO females mated with *Tfam* ^wt/Δ^ males. Columns represent mean ± S.E.M., and dots indicate independent trials. Differences were tested using a paired *t*‐test (*p < 0.05). (F) Genotype proportion of offspring of wt and *Tfam* cKO females mated with *Tfam* ^wt/Δ^ males. Stacked columns represent mean ± S.E.M. of genotypes in individual litter (number of litters is noted in brackets). A one‐sample t test was used to compare the recorded genotype ratio with a hypothetical value (i.e., 50%; **p < 0.01). (G) Implantation sites recorded in pregnancies of females mated with *Tfam* ^wt/Δ^ males. Columns represent mean ± S.E.M., and dots indicate independent trials. Differences were tested using a paired *t*‐test. Rate of abortion after mating of wt and *Tfam* cKO females with *Tfam* ^wt/Δ^ males. Stacked columns represent mean ± S.E.M. of embryos and abortus; number of pregnancies is indicated in brackets. Differences were tested using the Chi‐square test (****p < 0.0001). (H) The IVF outputs of *Tfam* ^wt/Δ^ compared to *Tfam* ^wt/wt^ littermates, both descendants of wt females. Columns represent mean ± S.E.M., and dots indicate independent IVF assays. Differences were tested using a paired *t*‐test (*p < 0.05).

Despite decreased oocyte viability observed in the previous experiment, we did not find any changes in the conception rate of cKO females mated with wt males (Figure 3A). However, the litter size of *Tfam* ^wt/Δ^ offspring was decreased (Figure 3B), although the oocyte yield was equal (Figure 3C). Due to possible embryonic loss, we recorded implantation sites and developing embryos beyond embryonic day E13.5. Obviously, the implantation capability of *Tfam* ^wt/Δ^ embryos is not impaired. However, these embryos are disadvantaged in fetal development (Figure 3D). Taken together, we can claim that cKO females are subfertile, but we wanted to assess the contribution of oocyte mitochondria. We designed the backcross of wt and cKO females with *Tfam* ^wt/Δ^ males, leading to (a) the production of *Tfam* ^wt/Δ^ offspring via fertilization of healthy eggs (wt females), and (b) the production of *Tfam* ^Δ/Δ^ genotype through the breeding of cKO females. Firstly, we similarly observed the reduced litter size of cKO females (Figure 3E); secondly, the genotype of *Tfam* ^Δ/Δ^ is not viable (Figure 3F); likewise, the loss occurs during post‐implantation embryonic development (Figure 3G). In contrast to a reduced litter size of *Tfam* ^wt/Δ^ (Figure 3B), we did not record any suppression of *Tfam* ^wt/Δ^ genotype in the reciprocal breeding (compare *Tfam* ^wt/wt^ and *Tfam* ^wt/Δ^ in Figure 3F). Accordingly, we assign this phenomenon to oocyte‐born mitochondrial insufficiency, rather than the nucleus‐encoded (i.e., TFAM‐derived) disability of mitochondrial biogenesis. Moreover, the fertilization ability of *Tfam* ^wt/Δ^ sperm is not impaired (Figure 3H).

### 3.4 *Tfam*‐Deficient Eggs Develop Into mtDNA‐Deficient Preimplantation Embryos

**FIGURE 4:**
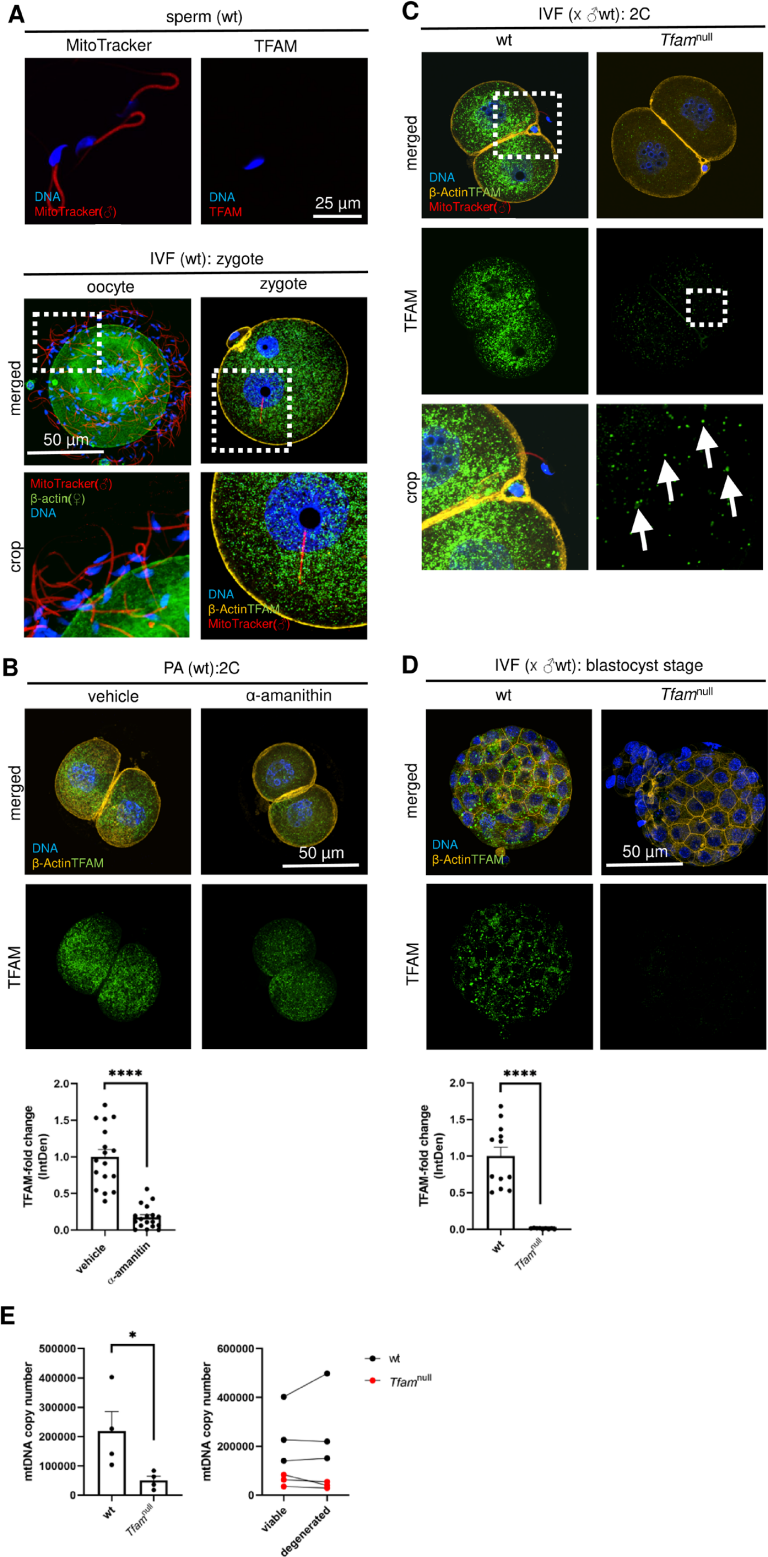
Different parental‐origin mitochondria staining and TFAM dynamics in early embryos after embryonic genome activation. (A) Vital mitochondria staining via MitoTracker Deep Red and the immunocytochemistry of TFAM in spermatozoa were then used for *vitro* fertilization (IVF). Differential staining of paternal and maternal mitochondria via fertilization with mitochondria‐labeled spermatozoa and the immunocytochemistry with the anti‐TFAM antibody, respectively. (B) Test of TFAM expression in wt two‐cell embryos after embryonic genome activation (EGA) via α‐amanitin, specific inhibitor of RNA polymerase II. (C) Maternal and paternal TFAM dynamics in two‐cell IVF embryos after EGA. Rectangles indicate emphasized areas; arrows point to TFAM loci in the cytoplasm. (D) The analysis of TFAM in IVF‐produced blastocysts. Columns represent mean ± S.E.M., and dots indicate individual blastocysts. Differences were tested using the unpaired *t*‐test (*p < 0.05; ****p < 0.0001). (E) Measurement of mtDNA copy number in IVF blastocysts and comparison of viable blastocysts to degenerated ones. Columns represent mean ± S.E.M., and dots indicate individual blastocysts; differences were tested using a paired *t*‐test (*p < 0.05).

Based on previous findings of mtDNA‐depleted eggs, we consider compensation of *Tfam* deficiency via expression of paternal *Tfam* allele after embryonic genome activation. Firstly, we were focused on distinguishing paternal and maternal mitochondria in wt zygotes, using differential mitochondrial staining (Figure 4A). Depiction of the fertilized egg (i.e., the zygote) and dynamics of mitochondrial biogenesis during early embryonic development. Using α‐amanitin, a specific inhibitor of RNA polymerase II, we assessed the contribution of embryonic genome activation (EGA) to TFAM abundance in post‐EGA two‐cell embryos (Figure 4B). Decreased TFAM signal in α‐amanitin wt embryos points out the expression of the embryonic *Tfam* gene; accordingly, we may assign TFAM spots in embryos produced by the IVF of *Tfam* ^null^ eggs with sperm to post‐EGA expression of paternal *Tfam* allele (Figure 4C). Accordingly, we expected the increasing necessity of *Tfam* expression as published earlier [24]; although we have evidence of *Tfam* expression in the early post‐EGA embryo (Figure 4B,C), there is no parent‐of‐origin compensation of TFAM abundance in blastocysts through the expression of paternal *Tfam* allele (Figure 4D), and we can assign the signal to the maternal pool from the egg. Similarly to the negligible signal of TFAM in blastocysts, there is a very low mtDNA copy number, corresponding to the mitochondrial genome inherited from the egg (Figure 4E). Above that, the mtDNA copy number does not significantly drive the success of embryonic development into viable embryos (Figure 4E).

### 3.5 Recovery of mtDNA Abundance Through Embryonic Mitochondrial Activation

**FIGURE 5:**
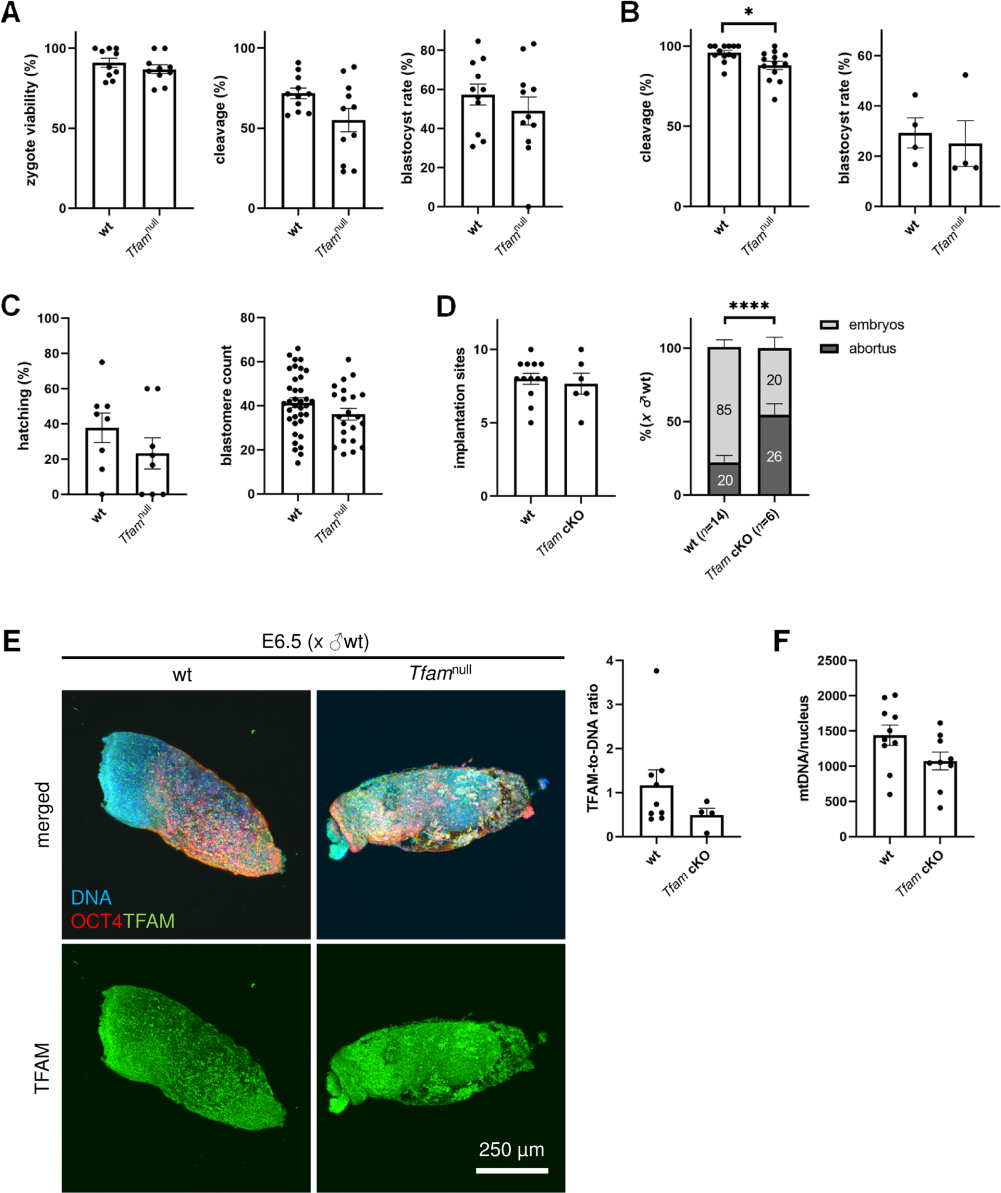
Success rate of *Tfam* ^wt/Δ^ and *Tfam* ^Δ/Δ^ embryos in vitro and mitochondrial status after implantation. (A) The outputs of the in vitro fertilization (IVF): Cleavage, blastocyst rate, and hatching rate. (B) Parthenogenetic activation (PA) and the success of early development of *Tfam* ^Δ/Δ^ embryos. Columns represent mean ± S.E.M., and dots indicate independent IVF/PA assays. Differences were tested using a paired *t*‐test (*p < 0.05). (C) Assessment of blastocyst quality was performed via blastomere count and hatching rate. Dots indicate independent blastocysts, and the line shows the median. Differences were tested using the unpaired *t*‐test. (D) Assessment of implantation ability of E6.5 embryos and rate of abortion after mating of wt and *Tfam* cKO females with wt males. Stacked columns represent mean ± S.E.M. of pregnancies or litters. Differences were tested using the Chi‐square test (****p ≤ 0.0001). (E) Analysis of TFAM in post‐implantation embryos at embryonic day E6.5. Signal density of TFAM is related to DNA signal stained with 4′6′‐diami‐dino‐2‐phenylindole (DAPI). (F) MtDNA copy number in *Tfam* ^wt/Δ^ and *Tfam* ^wt/wt^ E6.5 embryos. MtDNA is related to nuclear DNA, and the mtDNA is expressed as the ratio. Columns represent mean ± S.E.M., and dots show individual embryos. Differences were tested using a paired *t*‐test.

Previous experiments show that a fully developed preimplantation embryo in the blastocyst stage can develop independently of both *Tfam* expression and mtDNA abundance. However, we considered the impaired ΔΨm of mitochondria in the egg as a factor in the success of fertilization and further early development. For this reason, we used IVF with wt sperm for the tracking of developmental competence of *Tfam* ^null^ eggs (i.e., *Tfam* ^wt/Δ^ embryos), and we observed neither a decrease in zygote viability, cleavage nor blastocyst rate (Figure 5A). To avoid the paternal genome in early embryos, we used parthenogenetic activation and produced *Tfam* ^Δ/Δ^ embryos; then, we observed decreased cleavage of *Tfam* ^Δ/Δ^ embryos. However, once embryos were cleaved, their chance to achieve the blastocyst stage was not affected (Figure 5B). In addition to developmental competence, we did not observe any change in the hatching and blastomeres' count, and we suggest *Tfam* ^wt/Δ^ embryos are equally competent to achieve implantation (Figure 5C). Accordingly, the number of implantation sites is not affected. However, the proportion of abortus increased (Figure 5D). The mtDNA‐depleted blastocysts of *Tfam* ^wt/Δ^ genotype did not show a lower predisposition for implantation, and we suggest the effective bottleneck effect applies not earlier than when the embryo is implanted in the uterus. Concurrently, we assumed a recovery of the mitochondrial genome in survivor embryos, and therefore we tested the quality of post‐implantation embryos at embryonic day E6.5. Indeed, we did not observe a changed signal density of TFAM (Figure 5E). According to the renewal of *Tfam* expression, the mtDNA copy number achieves the level observed in wt embryos (Figure 5F). Due to this coupled awakening of *Tfam* transcription and mtDNA replication, we suggest “embryonic mitochondrial activation” after the embryo implantation, meaning the initiation of mtDNA biogenesis.

### 3.6 Adult Survivors Show Mitochondrial Health

**FIGURE 6:**
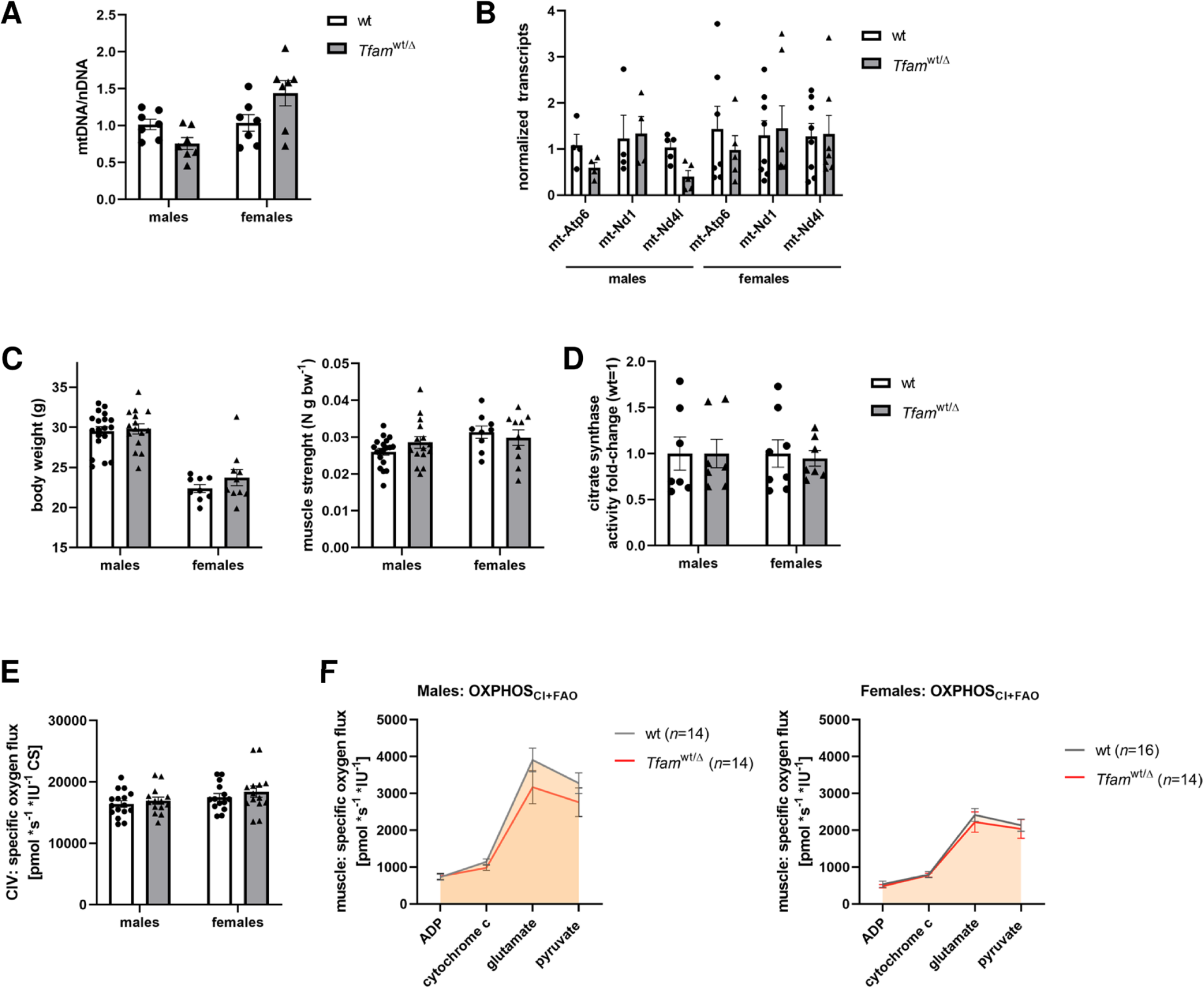
Evaluation of mitochondrial status in skeletal muscle of *Tfam* ^wt/Δ^ offspring. (A) The mtDNA copy number assessment in the skeletal muscle tissues of wt and *Tfam* ^wt/Δ^ offspring. mtDNA copy number was normalized to nuclear DNA. Differences between mean dCt values of wt and *Tfam* ^wt/Δ^ offspring were tested, using a paired *t*‐test. (B) Mitochondrial transcripts in muscle tissues. Gene expression levels were normalized to β‐actin expression; columns show mean ± S.E.M. of normalized fold expression values. Differences between mean dCt values of wt and *Tfam* ^wt/Δ^ offspring were tested, using a paired *t*‐test. (C) The body weight and test of muscle strength (N) related to g.bw^−1^. (D) Measurement of citrate synthase and electron chain transport of wt and *Tfam* ^wt/Δ^ (F1) muscle tissue. (E) Assessment of the activity of complex IV related to the activity of citrate synthase. Columns represent mean ± S.E.M., and dots indicate individual animals. Differences were tested using a *t*‐test. (F) Measurement of electron chain transport in muscle tissue of male and female offspring. Connecting lines show electron transport through complexes of oxidative phosphorylation. Differences were tested using a *t*‐test. The number of tested animals is indicated in brackets.

We considered the power of the post‐implantation bottleneck and tested the mitochondrial health of survivor embryos that developed into healthy individuals. We focused on muscle tissue, due to its high energy demand and, therefore, its sensitivity to mitochondrial failure [25]. Tracking both males and females separately, we did not observe any difference in mtDNA copy number (Figure 6A) or mtRNAs between wt and *Tfam* ^wt/Δ^ animals (Figure 6B). Furthermore, we also did not observe any difference in mtDNA copy number and mtRNA transcription in the liver, brain, and heart (data not shown). We performed the trial of muscle strength normalized to body weight, and we did not reveal any failure of *Tfam* ^wt/Δ^ genotype (Figure 6C). We proved the absence of mitochondrial dysfunction in *Tfam* ^wt/Δ^ males and females, using a biochemical assessment of citrate synthase activity (Figure 6D), activity of CIV complex (Figure 6E), and electron chain transport measurement (Figure 6F). Based on the results of the complex evaluation of muscle tissue of *Tfam* ^wt/Δ^ adults, we infer a post‐implantation bottleneck effect, ensuring the selection of mitochondrially healthy individuals.

## 4 Discussion

In this study, we used *Tfam* knockout to generate mtDNA‐depleted eggs and to test the necessity of maternal mitochondria for embryonic success. We observed a very low mtDNA copy number in fully grown oocytes, similarly to cells lacking mtDNA (termed rho cells) as described [26]. The copy number that we detected in *Tfam* ^null^ eggs corresponded to primary oocytes before the growth phase started [27]. Interestingly, regardless of the deficiency of mtDNA, *Tfam* ^null^ oocytes underwent growth, necessarily including synthesis and accumulation of housekeeping proteins, components of zona pellucida, and membrane organelles [28]. The observed amount of mtDNA copy number is in accordance with our model, created based on the excision of *Tfam* by Cre recombinase, whose expression is under control of the zona pellucida glycoprotein 3 (ZP3) promoter.

Although a decreased mtDNA copy number was presumed, based on previous findings [29], our experiment using transmission electron microscopy revealed that mitochondrial mass is unaffected in fully grown *Tfam* ^null^ oocytes. For this reason, we suggest most mitochondria are free of mtDNA. This observation points out the independence of mitochondrial fission from mtDNA replication during oocyte growth. Furthermore, as the mitochondrial membrane potential ΔΨm was significantly decreased without any observable changes in overall mitochondrial mass, we may conclude that mitochondrial autophagy is relaxed in the growing oocyte. Our observation of the reduced mitochondrial membrane potential ΔΨm in *Tfam* ^null^ oocytes was concurrent with a decrease in mtRNA transcription, which is assumably associated with the proper assembly of new electron transport chain complexes I, III, and IV, as well as the assembly of the ATP synthase [30].

Our assumption of relaxed autophagy in the growing *Tfam* ^null^ oocytes could represent a compensatory response to an “inferior” mitochondrial pool, as we had previously suggested [31]. The respective up and down regulation of autophagy in response to the size of the oocyte mitochondrial pool explains the observation that mtDNA copy number offers a protective effect against the accumulation of harmful mutations in the oocyte and aids purifying selection mechanisms by allowing upregulation of autophagy [12]. In addition to the oocyte‐born mtDNA mutagenesis, we can presume the zygote to be vulnerable to leakage of paternal mtDNA [32] due to damaged oocyte‐driven sperm mitophagy [33].

While we observed a slight decrease in oocyte viability associated with oocyte mitochondrial dysfunction, fertilization and development competence up to and including implantation was largely unaffected. Our findings support the current understanding of the energy need of oocytes. There is no doubt of the necessity for energy for the spindle assembly and chromosome segregation in the oocyte [2], but the oxidative phosphorylation requirement is kept to a minimum throughout oogenesis and preimplantation development [11, 34]. Regarding the non‐respiration roles of mitochondria, we consider mitochondrial contribution in the egg to the Ca^2+^ deposit needed for oocyte activation after sperm penetration [35, 36]. Accordingly, we observed no change in fertility rate, and obviously, mtDNA‐depleted mitochondria offer sufficient apparatus for the activation of *Tfam* ^null^ egg and zygote formation after fertilization with a spermatozoon.

Following the description and phenotyping of *Tfam* ^null^ eggs, we focused on the effect of deficient mtDNA on the success of embryonic development. Due to the redundant mitochondrial mass in the egg [4] and the silence of mtDNA biogenesis in early embryos [11], we assumed that *Tfam*‐deficient embryos would develop to the blastocyst stage. Indeed, we observed successful blastocyst achievement, capable of nidation to the endometrium. Our findings of decreased litter size were due to the higher abortus rate confirmed by a selective bottleneck after embryo implantation, as described earlier [4, 29]. However, the oocyte threshold of mtDNA for successful embryonic development seems to be lower (8231 ± 1.857 copies) than previously estimated (18 000 copies) [4]. In addition, we observed that mtDNA deficiency is not compensated for earlier than when the embryo is implanted, that is, the paternal allele of *Tfam* is not expressed in early embryos after EGA, and the mtDNA copy number does not reach physiological levels until the post‐implantation embryos. Due to this fact, the mtDNA copy number does not seem to be a suitable predictor of blastocyst quality usable for selection for embryo transfer, as previous findings indicated [13, 14].

Therefore, we were concerned about the mutation load and distribution of mtDNA variants into somatic tissues of embryos, further propagated within postnatal life [37]. Although mtDNA sequencing and its throughput in ontogeny are studied, we considered the general response to default mtDNA insufficiency. Thus, we assessed the mitochondrial health of offspring and focused on muscle tissue, mostly responding to mitochondrial insufficiency [25]. Surprisingly, neither males nor females of *Tfam* ^wt/Δ^ genotype show any decline in mitochondrial fitness, measured by mtDNA amount, mitochondrial transcriptional activity, mitochondrial respiration capacity, and muscle strength. We take into consideration the age‐related effect of mitochondrial decline due to the accumulation of mtDNA mutations [38]. Accordingly, a comprehensive investigation of the mitochondrial fitness of aging *Tfam* ^wt/Δ^ individuals is needed.

Taken together, our study provided evidence that the bulk of the oocyte maternal mtDNA genome is dispensable for the entire period of mouse oogenesis, following zygote formation and blastocyst achievement. We suggest the major role of the mitochondrial genome is the transmission of genetic information. The dispensability of mitochondria during early embryonic development provides a distinct advantage, as it allows the package of mtDNA into inactive nucleoids by TFAM, thereby simultaneously reducing the risk of introducing deletion events during replication [39], and presumably protecting the mitochondrial genome against oxidative damage during a period in the uterus where the embryo could be vulnerable to hypoxia [40]. Interestingly, there is a large plasticity in the mitochondrial genome abundance, where the growing oocytes reach variable amounts of mtDNA content, and consequently a fully grown oocyte can survive a deviation from the norm due to the physiological redundancy of mtDNA.

Obviously, mitochondrial genome activation in post‐implantation embryos constitutes the bottleneck preventing mitochondrial dysfunction from affecting the sensitive organogenesis during the fetal period or the postnatal life of descendants. On the other hand, the implantation bottleneck may be responsible for early pregnancy loss (e.g., in human IVF program; [41]) because a mitochondrial‐incompetent blastocyst is unable to develop beyond the later stages when the mitochondrial genome is activated [42].

## Author Contributions

M.S., M.V., J.J., J.C., Z.T., and J.N. designed the study. M.S., M.V., T.B., M.M., J.J., K.G., K.P., Z.T., G.P., and J.N. performed experiments. M.S., J.J., T.B., K.G., J.K., and J.N. analyzed data. M.S. and J.N. conceptualized and wrote the manuscript.

## Conflicts of Interest

The authors declare no conflicts of interest.

## Data Availability

The data that support the findings of this study are available in the Results of this article.
